# Potential Effects of Oilseed Rape Expressing Oryzacystatin-1 (OC-1) and of Purified Insecticidal Proteins on Larvae of the Solitary Bee *Osmia bicornis*

**DOI:** 10.1371/journal.pone.0002664

**Published:** 2008-07-16

**Authors:** Roger Konrad, Natalie Ferry, Angharad M. R. Gatehouse, Dirk Babendreier

**Affiliations:** 1 Agroscope Reckenholz-Tänikon Research Station ART, Zürich, Switzerland; 2 Institute for Research on Environment and Sustainability, School of Biology, University of Newcastle Upon Tyne, Newcastle, United Kingdom; University of Melbourne, Australia

## Abstract

Despite their importance as pollinators in crops and wild plants, solitary bees have not previously been included in non-target testing of insect-resistant transgenic crop plants. Larvae of many solitary bees feed almost exclusively on pollen and thus could be highly exposed to transgene products expressed in the pollen. The potential effects of pollen from oilseed rape expressing the cysteine protease inhibitor oryzacystatin-1 (OC-1) were investigated on larvae of the solitary bee *Osmia bicornis* ( = *O. rufa*). Furthermore, recombinant OC-1 (rOC-1), the Bt toxin Cry1Ab and the snowdrop lectin *Galanthus nivalis* agglutinin (GNA) were evaluated for effects on the life history parameters of this important pollinator. Pollen provisions from transgenic OC-1 oilseed rape did not affect overall development. Similarly, high doses of rOC-1 and Cry1Ab as well as a low dose of GNA failed to cause any significant effects. However, a high dose of GNA (0.1%) in the larval diet resulted in significantly increased development time and reduced efficiency in conversion of pollen food into larval body weight. Our results suggest that OC-1 and Cry1Ab expressing transgenic crops would pose a negligible risk for *O. bicornis* larvae, whereas GNA expressing plants could cause detrimental effects, but only if bees were exposed to high levels of the protein. The described bioassay with bee brood is not only suitable for early tier non-target tests of transgenic plants, but also has broader applicability to other crop protection products.

## Introduction

Genetically modified (GM) crops expressing proteins from *Bacillus thuringiensis* (Bt) are cultivated on a rapidly increasing acreage worldwide [1] since their commercialization in the mid 1990s, and, with the exception of cotton expressing both Bt and CpTI (serine protease inhibitor from cowpea), are the only insect-resistant GM plants to have been brought to the marketplace. However, various strategies based on the use of plant or animal derived genes are actively being pursued [2], [3]. A major concern raised in connection with the cultivation of transgenic plants is their potential to harm beneficial insects such as bees. By pollinating wild and cultivated plants, bees make a significant contribution to the functioning of natural ecosystems and to the human food supply [4]. Honey bees (*Apis mellifera* L.) are regarded as the most economically important pollinators of agricultural crops worldwide [5]. However, solitary bees (both wild and managed) also provide an important pollination service and their value for agriculture is increasingly being recognized [6]–[8]. This is particularly true for areas and crops where honey bees are absent or are inefficient pollinators, and under these conditions non-*Apis* bees can substantially enhance production [9], [10]. In the late 1950s, the use of non-*Apis* bees (i.e. the two solitary bees *Nomia melanderi* Cockerell and *Megachile rotundata* Fabricius) started to become important in commercial pollination. Since then, several solitary bee species (mainly members of the genus *Osmia*) have been developed into manageable crop pollinators [11], [12] and have often been found to contribute to higher crop yields than honey bees (e.g. [13]).

Direct effects of transgene products on bees will depend upon both exposure to the protein and its toxicity. When foraging on an insect-resistant GM crop, bees are potentially exposed to the insecticidal protein by consuming pollen and nectar. Nectar is known to contain mainly sugars [14], whereas pollen is known to contain up to 61% protein [15]. For instance, relatively high levels of the Bt toxin Cry1Ab were found in the pollen of the first generation transgenic maize Event176 (4–7 µg g−1 dry weight, [16]). Although all bee pollinators consume pollen to some degree, their susceptibility to a transgene product expressed in pollen may vary among taxa due to relevant differences in physiology, behavior and ecology. Besides differences in the susceptibility to insecticidal protein, bee taxa vary also in the amount of foreign protein the larvae are exposed to. Whereas young honey bee larvae are mainly fed with glandular secretions from adult bees and ingest only very small amounts of pollen [17], [18], larvae of solitary bees feed on provisions mainly consisting of pollen throughout their entire feeding period [19]. Thus, if the transgene is expressed in the pollen, the solitary bee larvae will be directly exposed.

Larvae are often regarded as the most vulnerable stage in an insect's life cycle. However, there are relatively few studies investigating potential effects of insect-resistant transgenic plants, or their purified transgene products, on honey bee larvae [20]–[23] and no such studies have been carried out to date for larvae of solitary bees. Toxicity to solitary bee larvae has been measured for pesticides, although mainly under field conditions [24]. When pollen provisions manually ‘spiked’ with test substances where used, the relative test concentrations were not standardized due to the addition of a defined absolute amount of test compounds to bee-collected pollen provisions of probably variable size [25]–[27].

Due to the increasing importance of Bt-expressing crops globally, Cry proteins isolated from the bacterium *B. thuringiensis* have been extensively studied in terms of their impact on beneficial insects. Various studies have assessed Bt-plants both in laboratory and field trials and revealed no adverse effects on honey bees or bumble bees [20], [28], [29]. The use of genes encoding lectins or protease inhibitors (PIs) represents two further strategies for the production of GM crops with enhanced levels of resistance to insect pests. Both groups of proteins have a relatively broad activity range. The snowdrop lectin (*Galanthus nivalis* agglutinin, GNA) has been shown to be effective against insect pests from several different orders [30], [31]. With respect to hymenopteran species, a GNA concentration of 0.1% has been found to cause direct toxic effects on bumble bees [32] and four different parasitic wasps [33]–[35]. Protease inhibitors interfere with digestion of dietary protein by specific binding to proteolytic enzymes within the insect gut. Inhibitors of the serine proteases trypsin and chymotrypsin have been found to increase bee mortality when provided at concentrations of 0.1% or more [32], [36], [37].

Despite their importance for seed setting in wild and cultivated plants, solitary bees have never been included in non-target tests of transgenic plants or purified transgene products. The aims of this study were therefore to: (i) develop suitable experimental methods for laboratory-based non-target testing of transgenic plants or purified transgene products and solitary bee larvae and (ii) investigate potential direct toxic effects of an insect-resistant transgenic crop or purified insecticidal proteins on life cycle parameters of a solitary bee species. The model system consisted of the red mason bee *Osmia bicornis* L. ( = *O. rufa*) (Hymenoptera: Megachilidae) and a transgenic oilseed rape (*Brassica napus* L.) expressing the cysteine protease inhibitor oryzacystatin-1 (OC-1) originating from rice (*Oryza sativa* L.). The insecticidal proteins (i) OC-1, (ii) GNA, and (iii) the Bt toxin Cry1Ab were tested in purified form by adding them to the pollen of control oilseed rape plants. Oilseed rape is not only a relevant forage plant for wild and managed bees because of its ample pollen and nectar, it is also a very important field crop in Europe and many other parts of the world [38]. *Osmia bicornis* is a univoltine, polylectic solitary bee prevalent in Europe and northern Africa [39]. It nests above-ground in pre-established cavities and readily accepts artificial nest devices [40], which greatly facilitates obtaining, inspecting and manipulating individual nest cells. Furthermore, *Osmia* sp. have been shown to efficiently pollinate caged mustard crops (Brassicaceae) [41].

## Materials and Methods

### Reagents

OC-1 was prepared as a recombinant *Escherichia coli*-produced protein (rOC-1) as previously described by Ferry et al. [42] whilst lyophilized insecticidal δ–endotoxin Cry1Ab (from *B. thuringiensis* var. *kurstaki*) was obtained from M. Carey (Dept. Biochemistry, Case Western Reserve University, Cleveland, USA). GNA was purchased from E. van Damme (Ghent University, Belgium) [43]. An ELISA (enzyme-linked-immunoabsorbant assay) kit for Cry1Ab was obtained from Agdia Inc, New Jersey, USA and HRP-conjugated goat anti-rabbit IgG from Bio-Rad Laboratories GmbH, München, Germany. Bovine Serum Albumin (BSA) and phenylmethylsulphonyl fluoride (PMSF) were purchased from Sigma.

### Plant material

Homozygous transgenic spring oilseed rape cv. Drakkar, line OC-1 Drakkar 4B expressing the anti-metabolic protein OC-1 [44], was grown from seeds; the non-transformed line Drakkar (isoline) was used as the control. Plants were grown in 1.8 L plastic pots in compost soil and cultivated in the glasshouse at 25±5°C, L16∶D8. They received one dose of the fertilizer Superwux 0.4% (N10∶P10∶K8) (Samen Mauser, Winterthur, Switzerland) six weeks after sowing.

### Determination of transgene expression in oilseed rape

The level of OC-1 expression in plant tissues was determined by dot-blot immunoassay. Fresh pollen and leaf samples were taken at random both from transgenic and non-transformed oilseed rape plants. For the pollen, fresh flowers were cut and the pollen was carefully removed from the anthers with a brush and tweezers, weighed, and transferred to a test tube. Leaf samples were lyophilized and ground to a fine powder, whilst pollen was directly ground in 100 mM Tris-HCl buffer, pH 7.5 (containing 1% PMSF; 36 mg ml−1 in ethanol), and incubated overnight at 4°C with shaking. Both leaf and pollen extracts were centrifuged at 10,000 g for 15 min and total soluble protein of the supernatant was estimated by Bradford assay using BSA as a standard [45]. Samples were subsequently diluted in phosphate buffered saline (PBS) to give a final protein concentration of 60 µg ml−1 for pollen extract and 20 µg ml−1 for leaf extract. In total, 60 µg pollen protein and 20 µg leaf protein were loaded onto 0.2 µm nitrocellulose in a Bio-Rad dot-blot apparatus. Purified rOC-1 was used to provide a set of standards (12.5, 25, 50, 100, 500 ng) with which to compare OC-1 expression in transgenic plants. The dot-blot assay was carried out via the standard procedure [46]. OC-1 was detected by enhanced chemiluminescence (ECL) as previously described [46], using polyclonal antibodies raised against OC-1 as the primary antibody (1∶2,500 dilution) and HRP-conjugated goat anti-rabbit IgG as the secondary antibody (1∶10,000).

### Experimental design

#### Production of bees

Adult *O. bicornis* of both sexes were purchased from Dr. Schubert Plant Breeding (Schwerz, Germany) in winter while hibernating in their cocoons. Bees were stored at 3±1°C, 65±10% RH and, when required, exposed to warm temperature (25±5°C) to initiate emergence from the cocoon.

Provision masses of solitary bees do not contain only pollen and nectar, but also specific gland secretions with assumed anti-bacterial and/or anti-fungal properties [19], [39]. In order to provide the developing larvae in the feeding assay with bee-produced pollen provisions the requirements of brood rearing by *O. bicornis* were met under standardized indoor conditions. Two cages (3.0×2.0×2.5 m) were installed in glasshouse units (25±5°C, L16∶D8) containing 30–70 flowering oilseed rape plants either of the transgenic OC-1 line or the non-transgenic isoline. Throughout the course of the experiment, withering plants were replaced by fresh ones. Each cage contained artificial nesting units (metal cans filled with paper tubes of 8 mm diameter) and the raw material for building nest cell partitions (approximately 1 kg of field-collected soil, kept moist). To ensure that nectar availability was not a limiting factor for cell provisioning and food supply was sufficient at all times, sugar solution (50% w∶v) was offered in bird feeders. Newly emerged bees of both sexes were released into the cages with a constant population of approximately 60 bees per cage (2∶1 F∶M sex ratio). After mating, the females started building linear series of up to four nest cells separated by mud partitions in the provided paper tubes. Each cell contained a loaf of pollen mixed with nectar, on top of which an egg was laid. Nesting units were checked daily for paper tubes with a freshly sealed entrance (indicating completed nesting activity) and these tubes were removed.

#### Treatments

Sealed paper tubes were carefully opened and the contents of the brood cells were extracted with forceps. Pollen masses originating from the transgenic plants were used to test for effects on larvae consuming transgenic OC-1 pollen. Masses consisting of pollen collected from non-transformed control plants were randomly assigned to either a treatment with purified insecticidal protein or to the control without protein additive. The insecticidal proteins tested were: rOC-1 (final concentration 0.1% of fresh pollen provision w∶w), GNA (0.01% and 0.1%), and Cry1Ab (0.01%) (Table 1). The concentration of 0.01% GNA was chosen to approximate a level of insecticidal protein that bees would potentially be exposed to in the field [47], [48] and the concentration of 0.1% rOC-1 and GNA, respectively, was chosen to represent an unrealistically high concentration (worst-case scenario). This also applied to 0.01% Cry1Ab since even plants with pollen-specific promoters contained less than 0.001% (w∶w) Cry1Ab in pollen (e.g. the Bt-maize Event176, [16]). To distribute the insecticidal protein within the pollen mass as evenly as possible without removing the attached egg, it was dissolved in water (2% and 0.2% w∶v, respectively) and 50 µl of this solution g−1 provision was delivered into a longitudinal fissure previously formed in the provision mass by a small metal spatula. The provision mass with the attached egg was then transferred to a round-bottomed glass vial (inside length 37 mm, inside diameter 10 mm) for the observation of larval development. The vial was positioned horizontally, sealed with a cotton plug and incubated under standardized conditions (20±1°C, 75±5% RH, no light).

**Table 1:** Experimental treatments of feeding assay with *Osmia bicornis* larvae.

Pollen type of provision	Additive	Concentration
Transgenic OC-1 pollen	H2O	0.004–0.009%a
	rOC-1	0.1%
	GNA	0.01%
Pollen from isoline	GNA	0.1%
	Cry1Ab	0.01%
	H2O	0% (control)

Larval pollen provisions were collected and processed by nesting bees. Provisions containing non-transgenic pollen from the isoline were manually ‘spiked’ with insecticidal protein dissolved in water to achieve the test concentrations indicated (w∶w). aPercentage OC-1 of total soluble protein in pollen extract, determined by dot-blot immunoassay. Abbreviations: OC-1, oryzacystatin-1; rOC-1, recombinant OC-1; GNA, *Galanthus nivalis* agglutinin.

#### Measurements

The fresh pollen provision masses with the attached eggs were weighed on a microbalance (Mettler Toledo, MX5, d = 1 µg; ±2 µg, also used for all following weight measurements). Larval development was observed daily until cocoon spinning by the fully-grown larva impeded direct observations during the prepupal dormant stage, pupation and subsequent wintering. Larval body weight was recorded when the larvae had consumed all the available pollen provision. On day 120 after the application of insecticidal protein, bees underwent a 15-day pre-wintering period (14±1°C, 85±5% RH, no light) before entering the 150-day wintering period (3±1°C, 65±10% RH, no light). After wintering (i.e. 285 days after application of insecticidal protein), cocoons were removed from the glass vials, weighed, individually caged in 1.3 L transparent polystyrene straight-side containers (diameter 110 mm, height 160 mm, purchased from Semadeni AG, Ostermundigen, Switzerland) covered with a plastic lid with mesh and incubated in the glasshouse at 25±5°C, 16L∶8D. Cocoons were checked daily for emergence of adult bees which from then on received water but no food. Survival was recorded daily and dead bees were frozen at −20°C until lyophilized to determine dry weight. The cocoons from which no bee emerged within 30 days of incubation at warm temperature were dissected to confirm the bee's death and determine its sex.

Larval development time was measured as the number of days from hatching to the onset of cocoon formation and food conversion as the ratio between the body weight of the mature larva and the fresh weight of its provision mass. Relative weight loss during wintering was defined as the difference between the body weight of the mature larva and the weight of the adult bee and its cocoon at the end of wintering relative to the larval weight. Post-emergence longevity in spring represented the number of days a bee survived after successfully emerging from the cocoon.

### Stability of insecticidal protein over time (ELISA)

To test whether the larvae feeding on pollen provisions with added insecticidal protein were continuously exposed to these proteins throughout development, their concentrations in the provisions were determined over time by ELISA. For each of the four treatments with added insecticidal protein (Table 1), three provision masses without egg were normally treated with protein solution. In contrast to the provision masses in the feeding assay, these provisions were thoroughly mixed in Eppendorf tubes. Provisions were then incubated under the same conditions as the developing larvae (20±1°C, 75±5% RH, no light) and samples were taken at three different time points: immediately after the application of insecticidal protein (day 0), 14 and 28 days after application (days 14 and 28, respectively). Samples from pollen provisions were extracted as described above for plant tissues. Extracts were centrifuged at 13,000 g for 5 min and total soluble protein of the supernatants was determined by Bradford assay using BSA as a standard [45].

Extracts of Bt-treated provision samples were diluted in PBS+0.01% Tween 20 (v∶v) (PBST) to give a final protein concentration of 10 ng ml−1. From the batch of purified Cry1Ab toxin used in the feeding bioassay, a set of toxin standards was produced (final concentrations ranging from 0–10 ng ml−1 PBST buffer). The Cry1Ab ELISA kit was used according to the manufacturer's instructions. After the addition of the stop solution (H_2_SO_4_), absorbance was read at 450 nm in a microtitre plate reader. Levels of Cry1Ab were estimated from the Cry1Ab calibration curve. All incubations were performed in triplicate at room temperature.

Extracts from rOC-1 and GNA-treated pollen provisions were diluted in PBS to give a final protein concentration of 40 µg ml^−1^. From the batches of purified rOC-1 and GNA used in the feeding bioassay, a set of standards was produced (concentrations ranging from 0–1.2 µg ml−1 PBS buffer). ELISA plates coated with poly-clonal antibodies raised in rabbit against rOC-1 or GNA were used and the assays were conducted as described by Ferry et al. [42], except that the sera of primary and secondary antibody were diluted 1∶5,000. All incubations were performed in triplicate at room temperature.

### Data analysis

When necessary, data were transformed to meet the assumptions of parametric statistics. All statistical analyses were conducted using Statistica software (version 7.1, StatSoft Inc, Tulsa, USA). Mortality during larval development and during the later stages (i.e. prepupa, pupa and wintering imago) were first analyzed separately, and then also combined, using 2×2 contingency tables and Fisher's exact test. Larval development time and post-emergence longevity were analyzed by Cox proportional hazard models (log-likelihood test) including provision weight or larval body weight, respectively, as covariate [49]. Initiation of cocoon spinning represented the event in the analysis of development time whereas death was the event in the analysis of post-emergence longevity. Five pair-wise comparisons (i.e. each treatment with the control) were performed and significance levels were adjusted according to the sequential Bonferroni-Holm procedure [50]. Fresh weight of pollen provisions and food conversion data were analyzed using two-way analysis of variance (ANOVA) with treatment and sex as categorical predictors. Data for relative body weight loss during wintering were analyzed by one-way ANOVA excluding individuals that had successfully pupated but died during winter. Dunnett's test was used for post-hoc analysis, i.e. to compare each treatment with the control.

## Results

### Transgene expression in oilseed rape

**Figure 1:**
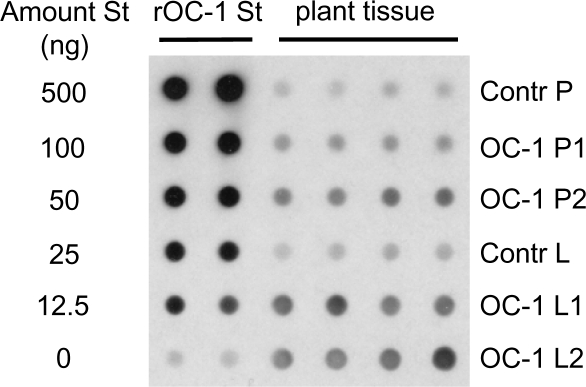
OC-1 expression in transgenic oilseed rape. Dot-blot immunoassay showing the expression level of the transgene product oryzacystatin-1 (OC-1) in leaf tissue and pollen of transgenic oilseed rape compared to non-transgenic oilseed rape (Contr). Lanes 1–2, range of recombinant OC-1 standards (St) assayed in duplicate; lanes 3–6, plant tissue extracts assayed in quadruplicate, two separate samples for transgenic OC-1 pollen (P) and leaves (L).

OC-1 expression was confirmed in leaves of the transgenic oilseed rape line and ranged from 0.047 to 0.074% of total soluble protein. Expression of OC-1 was also detected in the pollen ranging from 0.004 to 0.009% of total soluble protein, an order of magnitude lower than in vegetative tissues (Fig. 1).

### Effects of treatments on *O. bicornis* life history parameters

As eggs were randomly assigned to treatments, their sex was unknown and could only be determined retrospectively from those individuals which completed pupation. It was found that the proportions of females and males were not significantly different among treatments (Pearson χ^2^-tests; p>0.1 for all pair-wise comparisons). Furthermore, the fresh weight of pollen provisions did not significantly differ between treatments (two-way ANOVA; F_5,150_ = 0.954; p = 0.448; n = 20–43), whereas male larvae had significantly smaller provisions than female larvae (two-way ANOVA; F_1,150_ = 18.1; p<0.01; n(m) = 120; n(f) = 42).

#### Larval development, mortality and longevity after emergence

**Figure 2:**
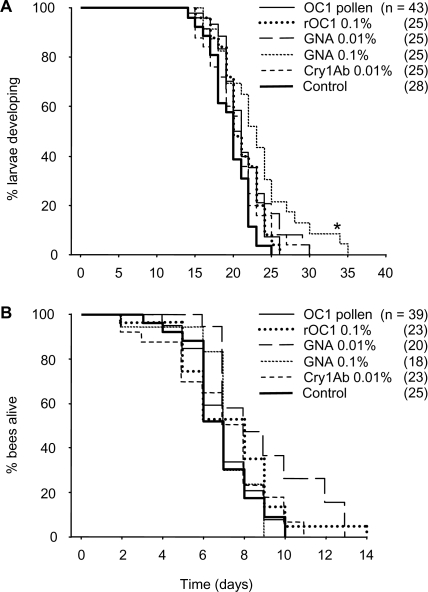
Development and longevity. Larval development time (days from hatching to mature larva) (A) and longevity as adults after emergence from the cocoon (B) of *Osmia bicornis* reared on transgenic OC-1 expressing oilseed rape pollen or on control pollen ‘spiked’ with insecticidal protein. Cox proportional hazard models followed by the Bonferroni-Holm procedure were used for both analyses. Significant differences from the control are indicated by asterisk. Abbreviations: OC-1, oryzacystatin-1; rOC-1, recombinant OC-1; GNA, *Galanthus nivalis* agglutinin.

Male and female larvae did not differ in development time (one-way ANOVA; F1,150 = 0.405; p = 0.525) and therefore, were combined in the subsequent analysis, which revealed significant differences among treatments (Cox proportional hazard model; χ^2^ = 27.5; df = 2; p<0.001; Fig. 2A). Development was significantly prolonged in larvae fed pollen with the highest level of GNA (0.1%) when compared to the control (padjusted = 0.002).

Of the 174 larvae tested, 5 (2.9%) died during larval development with a maximum of two within any one treatment (Table 2). Neither the transgenic OC-1 expressing pollen nor any of the treatments with added insecticidal protein differed significantly from the control in the number of dead larvae during the course of the bioassay (Fisher exact; p>0.1 for all pair-wise comparisons). Of the 169 individuals that successfully completed larval development, 17 (10.1%) died in subsequent stages, i.e. before emergence from the cocoon in spring, with a maximum of four within any one treatment. Also during this time span, no treatment differed significantly from the control in mortality (Fisher exact; p>0.1 for all pair-wise comparisons). These same results were obtained when the two observation periods were combined (Fisher exact; p>0.2 for all pair-wise comparisons).

**Table 2:** Bee mortality.

Treatment	During development	In cocoon	Combined
OC-1 pollen	0.00 (0.00 to 8.04)	9.09 (2.53 to 21.7)	9.09 (2.53 to 21.7)
rOC-1 0.1%	0.00 (0.00 to 13.2)	7.69 (0.95 to 25.1)	7.69 (0.95 to 25.1)
GNA 0.01%	3.85 (0.10 to 19.6)	16.0 (4.54 to 36.1)	19.2 (6.55 to 39.4)
GNA 0.1%	8.00 (0.98 to 26.0)	17.4 (4.95 to 38.8)	24.0 (9.36 to 45.1)
Cry1Ab 0.01%	0.00 (0.00 to 13.7)	8.00 (0.98 to 26.0)	8.00 (0.98 to 26.0)
Control	7.14 (0.88 to 23.5)	3.85 (0.10 to 19.6)	10.7 (2.27 to 28.2)
Total	2.87 (0.94 to 6.58)	10.1 (5.97 to 15.6)	12.6 (8.10 to 18.5)

Mortality of *Osmia bicornis* (in %) observed during development (i.e. from hatching to mature larva), in the cocoon (i.e. from start of cocoon spinning to emergence from cocoon) and over the whole observation time (n = 25–44). The 95% confidence interval is given in brackets. Abbreviations: OC-1, oryzacystatin-1; rOC-1, recombinant OC-1; GNA, *Galanthus nivalis* agglutinin.

Male and female bees did not differ in longevity after emergence from the cocoon (one-way ANOVA; F1,146 = 0.720; p = 0.398) and therefore, were combined in the same analysis. Post-emergence longevity of successfully over-wintered bees did not significantly vary among treatments (Cox proportional hazard model; χ^2^ = 17.7; df = 2; p = 0.054; n = 148; Fig. 2B). There was a weak although statistically significant positive correlation between post-emergence longevity and larval body weight (Pearson's correlation; r2 = 0.123; p<0.01; n = 132).

#### Food conversion and relative weight loss

**Figure 3:**
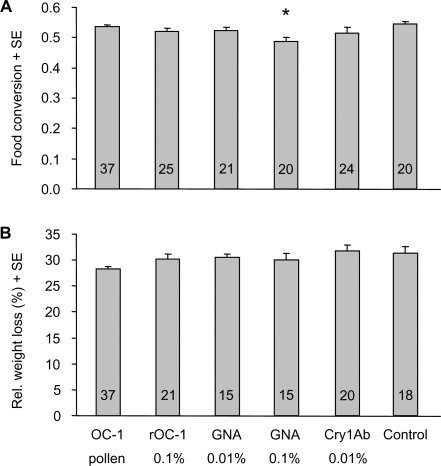
Food conversion and weight loss. Efficiency of converting the available provision into larval body weight (two-way ANOVA; Dunnett's test) (A) and relative body weight loss during wintering (one-way ANOVA; Dunnett's test) (B) of *Osmia bicornis* reared on transgenic OC-1 expressing oilseed rape pollen or on control pollen ‘spiked’ with insecticidal protein. Bars represent group means (+SE) and the enclosed figures the number of replicates. Significant differences from the control are indicated by asterisk. Abbreviations: OC-1, oryzacystatin-1; rOC-1, recombinant OC-1; GNA, *Galanthus nivalis* agglutinin.

Significant differences in food conversion were found between treatments (two-way ANOVA; F_5,140_ = 2.98; p = 0.014; Fig. 3A) and between male and female larvae (two-way ANOVA; F_1,140_ = 5.74; p = 0.018). Male larvae had a significantly lower food conversion than female larvae (mean(m) = 0.516; SE(m) = 0.006; n(m) = 103; mean(f) = 0.540; SE(f) = 0.007; n(f) = 44). In larvae that had received 0.1% GNA in their pollen provisions, food conversion was significantly reduced when compared to the control (Dunnett's test; p = 0.002), whereas this parameter was not significantly different from the control in the other treatments. There was a significant negative correlation between food conversion and larval development time (Pearson's correlation; r2 = 0.152; p<0.01; n = 145).

Analysis of relative body weight loss during wintering revealed small significant differences among treatments (one-way ANOVA; F_5,120_ = 2.50; p = 0.034; Fig. 3B). However, no treatment differed significantly from the control (Dunnett's test; p>0.062 for all pair-wise comparisons). Including sex as additional categorical predictor in the analysis did not significantly improve the model indicating that males and females did not differ in relative body weight loss.

### Stability of insecticidal protein over time

Immunoassay by ELISA was performed on pollen provision samples treated with insecticidal proteins to determine their stability at three different time points after protein application (0, 14 and 28 days). The results showed that high levels of these proteins could be detected in pollen provisions throughout the period of larval feeding (Table 3). After 28 days of incubation, however, all samples from provisions ‘spiked’ with rOC-1 contained at least 20% less insecticidal protein than detected initially and a relatively low level of Cry1Ab was measured in one of the three provision masses treated with this toxin. On average, larvae required 21 days to consume their pollen provisions. At this time, the remaining concentrations of rOC-1, GNA and Cry1Ab were approximately 75%, 93% and 85%, respectively, assuming a linear decrease from the sample means on day 14 to the sample means on day 28. In all extracts of provisions containing 0.01% GNA, the toxin concentration was below the detection limit of this assay.

**Table 3:** Stability of insecticidal proteins over time when added to pollen provisions.

Treatment	% of initial amount	
	*Day 14*	*Day 28*
rOC-1 0.1%	52.5 to 100 (83.7)	50.7 to 79.0 (66.5)
GNA 0.1%	77.7 to 100 (94.3)	75.3 to 100 (91.8)
Cry1Ab 0.01%	73.8 to 100 (94.7)	33.1 to 100 (75.3)

Amount of insecticidal protein determined by ELISA after 14 and 28 days of incubation presented as percentage of the amount detected immediately after protein application. Minimum and maximum values of independent determinations and the sample means (in brackets) are given (n = 3–9). Abbreviations: rOC-1, recombinant oryzacystatin-1; GNA, *Galanthus nivalis* agglutinin.

## Discussion

Due to their significant ecological and economic importance as pollinators, bees play a key role in non-target testing of insect-resistant transgenic crops. The present study is the first to investigate potential non-target effects of an insect-resistant transgenic crop on a solitary bee species.

In the present study, mortality was unaffected across all treatments, although sub-lethal effects were detected on larval development time and on the efficiency of converting pollen food into larval body mass when *O. bicornis* larvae were fed with 0.1% GNA. Furthermore, neither the transgenic OC-1 expressing pollen nor any of the other treatments with insecticidal protein (rOC-1 0.1%, Cry1Ab 0.01%, GNA 0.01%) had a negative impact on bee performance during larval development or subsequent pupation and wintering. Thus the impact of GNA on *O. bicornis* larvae appears to be dose dependent, in agreement with the results obtained by Babendreier et al. [32] in the only other published study that tested effects of GNA on bees. In that study, when bumble bee micro-colonies received GNA dissolved in sucrose solution, adult workers and males suffered from sharply increased mortality at 0.1% GNA, whereas less severe sub-lethal effects were found at 0.01%. With respect to the more pronounced effects observed by Babendreier et al. [32] compared to the present study, it should be noted that adult bumble bees and solitary bees consume much larger quantities of nectar (or sucrose solution) than pollen, although quantitative data for this are missing. This means that for a given test concentration, the absolute amount of toxin ingested by adult bumble bees in the study of Babendreier et al. [32] was approximately five to ten times greater than the amount ingested by *O. bicornis* larvae in the present study. Thus a greater impact on bee performance would have been anticipated in the study on bumble bees. Interestingly, in a different study where GNA was provided via pollen to bumble bee micro-colonies, neither adult nor larval mortality was affected in the 0.1% GNA treatment (Babendreier and Konrad, unpublished), an observation in agreement with the present study and more ecologically relevant as nectar generally does not contain proteins.

While no acute toxicity was observed in the present study, the sub-lethal effects on larval development observed at 0.1% GNA could negatively affect bee survival or fitness under field conditions. An extended larval development time may entail a higher risk of unsuccessful development due to longer exposure to potential hazards such as pathogen infection, parasitism or unfavorable weather conditions. In bees, as in most insects, offspring body size is strongly influenced by the amount of food ingested [51]; a negative impact on the efficiency of food conversion may lead to smaller individuals. This may adversely affect a bee population as body size has often been related to fitness in solitary bees and wasps [52].

Protease inhibitors have the potential to adversely affect insects by interfering with digestive proteolysis [53]. In the present study, the transgenic OC-1 expressing oilseed rape plants were found to produce OC-1 in the pollen at levels up to 0.009% of total soluble protein and neither the ingestion of this transgenic pollen nor of purified rOC-1 (0.1%) adversely affected *O. bicornis* larvae. To date, published information on expression of OC-1 or any other PI in the pollen of transgenic crop plants is scarce and when pollen of a transgenic PI expressing oilseed rape was analyzed, no PI was detected [48]. Thus relating the results of the present study to the concentrations of transgene PIs to which bees might be exposed to in the field remains difficult. However, it appears that a higher transgene expression level than the one observed in our OC-1 expressing model plant would be necessary for effective pest resistance [48]. Even when OC-1 was ingested at a concentration which is likely to exceed the level bees would be exposed to in the field, *O. bicornis* larvae remained unaffected. This is in agreement with a study where no short-term mortality was observed when providing young worker honey bees with a sucrose solution containing OC-1 at concentrations 20 times higher than in our high dose treatment [54], based on the assumption that proteins represent about one third of pollen dry mass in oilseed rape [15]. In contrast to a cysteine PI like OC-1, PIs that specifically bind to serine proteases (e.g. Kunitz Soybean Trypsin Inhibitor SBTI, Bowman-Birk Trypsin Inhibitor SBBI) have been found to significantly affect adult honey bees and bumble bees [21], [32], [36], [37], [55], [56], probably because serine proteases predominate in the digestive tracts of these bees [57], [58] and ingesting inhibitors of these enzymes can lead to quantitative and qualitative alterations in the digestive proteolysis [54], [56], [59]. The observed lack of impact of OC-1 on *O. bicornis* larvae may be due to a negligible role of cysteine protease activity or to adaptive changes in the protease profile in response to OC-1 ingestion. Very little is known about the digestive protease profiles of solitary bee larvae, but the results of the present study would indicate that the field cultivation of an OC-1 expressing transgenic crop is unlikely to be hazardous to *O. bicornis* larvae.

In the present study a Bt toxin concentration of 0.01% was shown to have no effect on *O. bicornis* life history parameters. This test concentration represents a worst-case scenario since it is at least one order of magnitude greater than the concentration observed in pollen of transgenic maize expressing the Bt toxin under the control of a pollen-specific promoter (Event176, [16]). Our results are in agreement with studies on potential effects of Cry toxins on honey bees [20], [29] and bumble bees [32], [60], but only few of these studies included measurements on the larval stages [22].

The risk that the cultivation of insect-resistant transgenic plants may pose for larvae of solitary bees is not only defined by the toxicity of the foreign protein (hazard) but also by the total amount of transgene product ingested (exposure). Exposure depends on the expression level of the insecticidal compound in the pollen, the amount of transgenic pollen present in the larval provision, and the persistence of the insecticidal protein in an active form over time. We have shown that the purified insecticidal proteins added to bee-collected pollen provisions could be detected at high levels beyond the mean larval development time. The stability of OC-1 and GNA to both high temperature and low pH, as well as the resistance of GNA to proteolytic digestion, have previously been reported [43], [61], [62]. Furthermore Cry1Ab is apparently not degraded in Bt maize pollen [16].

The amount of transgenic pollen present in the larval provision is largely influenced by (1) the attractiveness of the crop to bees, (2) the concurrence of the bloom of the crop in question and the bee's flight season, and (3) the distance of the nest from the relevant field. Oilseed rape is known to be very attractive to a number of social and solitary bees as it provides an abundance of nutritious pollen and nectar [38]. In the present study, foraging bees were confined to oilseed rape for the provisioning of their nest cells. In the case of field-grown transgenic oilseed rape, such severely restricted foraging would represent a worst-case scenario delivering the highest possible exposure to the transgene product. However, when other pollen sources are scarce in an area, native bees can rely almost exclusively on the abundant forage provided by flowering oilseed rape fields. *Osmia bicornis* females can restrict their visits to the same major pollen source (*Ranunculus* or *Quercus*) during their entire nesting period [63] and up to 40% of *O. bicornis* provision masses were found to consist exclusively of oilseed rape pollen [64]. The nesting period of *O. bicornis* coincides with oilseed rape bloom [65] and this bee, like most solitary bees, is known to have a relatively short foraging distance of only several hundred meters [66]. Thus, if an insect-resistant transgenic oilseed rape expressing the resistance trait in the pollen is grown in the field, larval provisions in nearby *O. bicornis* nests may contain relatively large proportions of the transgene product.

Since larvae of solitary bees feed almost exclusively on pollen [19], whereas young honey bee larvae receive mainly glandular secretions but only very small amounts of pollen [17], [18], solitary bee larvae are more likely to be exposed to potentially insecticidal proteins expressed in pollen. Furthermore, the impact of hazardous larval food on solitary bee populations could be severe, since many solitary bees have a relatively short reproductive season and only one offspring generation per year. With respect to the fact that biosafety studies have predominantly focused on the honey bee as a non-target insect pollinator [20], it is important to be cautious when extrapolating results with honey bees to solitary bees [67]. The significantly higher exposure of solitary bee larvae to transgene products expressed in pollen should be considered when defining the test concentrations for early tier experiments in an environmental risk assessment of transgenic plants and bees.

The experimental methods described here are not only suitable for early tier testing in non-target risk assessments of transgenic plants, but could also be adopted for pre-release laboratory testing of agrochemicals, including systemic pesticides which may be found in the pollen. In *in vitro* toxicity tests with honey bee larvae, high levels of control mortality, probably due to grafting, can be a problem [21], [23]. In the present study, however, low control mortality was observed which is likely to be due to the minimal handling of eggs and larvae required when rearing solitary bees on their provisions. As a further merit of the experimental design developed in this study, not only the larval rearing but also the pollen collection and preparation were conducted under standardized conditions which is an improvement compared to earlier studies investigating potential effects of pesticides on larvae of solitary bees [25], [26].

### Conclusions

The results of the present study indicate that it is very unlikely that either OC-1 or Cry1Ab would pose a risk to larvae of *O. bicornis*. GNA, whilst hazardous to *O. bicornis* at high dose (0.1%), was shown to not have any detrimental effect at expected expression levels (0.01%). These results obtained for *O. bicornis* as model species may be relevant for a large portion of the approximately 700 solitary bee species assumed to occur in Central Europe since many of them are also polylectic [39], forage on agricultural crops and reproduce during the bloom of such crops [7], [64].
